# Temperament and Character Traits of Female Eating Disorder Patients with(out) Non-Suicidal Self-Injury

**DOI:** 10.3390/jcm9041207

**Published:** 2020-04-22

**Authors:** Tinne Buelens, Koen Luyckx, Margaux Verschueren, Katrien Schoevaerts, Eva Dierckx, Lies Depestele, Laurence Claes

**Affiliations:** 1 Faculty of Psychology and Educational Sciences, KU Leuven, 3000 Leuven, Belgium; koen.luyckx@kuleuven.be (K.L.); margaux.verschueren@kuleuven.be (M.V.); lies.depestele@azt.broedersvanliefde.be (L.D.); laurence.claes@kuleuven.be (L.C.); 2 UNIBS, University of the Free State, 9300 Bloemfontein, South Africa; 3 Psychiatric Hospital Alexianen Zorggroep Tienen, 3300 Tienen, Belgium; katrien.schoevaerts@azt.broedersvanliefde.be (K.S.); eva.dierckx@vub.be (E.D.); 4 Department of Psychology and Educational Sciences, Vrije Universiteit Brussel, 1050 Elsene, Belgium; 5 Faculty of Medicine and Health Sciences, Universiteit Antwerpen, 2610 Wilrijk, Belgium

**Keywords:** non-suicidal self-injury, temperament, eating disorder, adolescence

## Abstract

Eating disorder (ED) patients show alarmingly high prevalence rates of Non-Suicidal Self-Injury (NSSI). Adolescents seem to be particularly at risk, as EDs and NSSI both have their onset in mid-adolescence. It has been suggested that personality could be a transdiagnostic mechanism underlying both EDs and NSSI. However, little attention has been given to adolescent clinical samples compared to adult and/or community samples. Therefore, the current study investigated the role of personality in a sample of 189 female inpatients with an ED (M = 15.93, SD = 0.98). Our results confirmed the high prevalence of NSSI in EDs, specifically in patients with bingeing/purging behaviours (ED-BP). Temperamental differences were found between ED-BP and the restrictive ED subtype (ED-R). Namely, ED-BP patients showed more harm avoidance and less self-directedness compared to ED-R. Temperamental differences were found in NSSI as well, regardless of ED subtype: ED patients who had engaged in NSSI during their lifetime reported less self-directedness and more harm avoidance. Interestingly, only ED patients who recently engaged in NSSI showed less novelty seeking. These temperamental profiles should be recognised as key mechanisms in the treatment of adolescent ED patients with and without NSSI.

## 1. Introduction

Non-Suicidal Self-Injury (NSSI) is defined as any socially unaccepted behaviour through which individuals deliberately and directly injure their own body [1]. NSSI can serve a wide variety of functions to the individual, but the most common self-reported function is to regulate negative emotions [2]. Typical methods of engaging in NSSI are cutting, scratching, burning, or bruising one’s own skin [3]. NSSI is strongly associated with internal distress, rejection by peers, rumination, and psychopathology in general, both internalising and externalising symptoms [4,5,6]. Adolescents seem to be most vulnerable, as NSSI onset peaks in mid-adolescence, around the ages of 14 and 15 [7]. The vulnerability of adolescents is also reflected in NSSI prevalence rates; epidemiological research consistently indicates that as many as 17% of adolescents in non-clinical samples have engaged in NSSI at least once [8,9,10]. Prevalence rates rise even higher in clinical samples, with young patients with an eating disorder being particularly at risk as up to 60% of this adolescent population engages in NSSI throughout their lifetime [11,12,13,14].

Eating disorder (ED) symptomatology, just like NSSI, can be considered a dysfunctional coping strategy to regulate negative affect [15,16]. Both in NSSI and EDs, the body is often perceived as negative and can become the target of emotional dysregulation [17]. EDs include Bulimia Nervosa (BN), characterised by recurrent binge-eating and compensatory behaviours (e.g., purging), and Anorexia Nervosa (AN), characterised by an irrational fear of gaining weight and restricting food intake. By definition, BN patients are at a healthy weight or slightly overweight, whereas AN patients are underweight [18]. However, some underweight patients do show bulimia-like symptoms, such as binge eating and purging. Consequently, AN is further divided into two subtypes: the binge-eating/purging subtype (AN-BP) and the restrictive subtype (AN-R).

A 2007 review [16] reported that AN-BP patients were most likely to engage in self-harming behaviour, with prevalence rates between 27.8% and 68.1% for this subtype. AN-R patients showed the lowest occurrence with prevalence rates between 13.6% and 42.1%. BN patients were in between the two AN subtypes, with self-harm prevalence rates ranging from 26% to 55.2%. However, this review investigated self-harm, which typically includes suicidal behaviour, in contrast to NSSI, which explicitly excludes suicidal thoughts and behaviours. Yet, several other studies have affirmed that AN-BP patients are engaging more frequently in NSSI compared to AN-R patients [19,20,21]. For instance, in a sample of 226 female ED patients, the lifetime prevalence of NSSI was significantly higher in AN-BP patients compared to AN-R patients [22]. Some uncertainty remains, however, as a handful of studies did not find any significant differences between AN-BP and AN-R [16,23]. Furthermore, research is lacking regarding the NSSI methods that are used by subtypes of ED patients. To the best of our knowledge, only four studies investigated differences in NSSI methods by subtypes of ED patients. Three of these studies did not find any significant difference [19,24,25], whereas one study by Claes et al. [26] reported that cutting was more common in female ED patients with binge/purge behaviour (AN-BP, BN) as compared to those with restrictive behaviour.

To improve understanding regarding the interplay between EDs and NSSI, researchers have been looking for transdiagnostic mechanisms. It has been suggested that personality could be such a transdiagnostic mechanism, underlying both EDs and NSSI [20,27,28]. According to the psychobiological theory of Cloninger et al. [29,30], there are seven dimensions of personality: four genetically determined temperamental dimensions and three learned character dimensions. Novelty seeking (NS) is the first temperamental dimension and refers to curiosity, impulsivity, enthusiasm regarding new experiences, and rash decision-making. Second, harm avoidance (HA) is characterised by shyness, fearfulness and inhibition in social situations. For example, one HA item reads: “When I have to meet a group of strangers, I am more shy than most people” [31]. Moreover, HA is characterised by excessive worrying, insecurity, and pessimism, even in situations where others would not worry or fear. Third, reward dependence (RD) describes seeking out social approval and support, openness to others, and a tendency to respond to signals of reward. Fourth, persistence (PS) refers to a competitive spirit, being inclined towards perfectionism, and showing perseverance in spite of repeated setbacks, frustration, or fatigue. Self-directedness (SD), the first of three character dimensions, is the strong desire to achieve a set of goals and values and the ability to regulate and adapt one’s own behaviour to reach these goals. Cloninger described SD as “willpower” [32], more recent literature linked SD to effortful control [33]. Second, cooperativeness (CO) concerns empathy, tolerance, agreeableness, and identification with others. Low CO is associated with a wide range of personality disorders [30]. Third and finally, self-transcendence (ST) involves spirituality and the idea of a transcendental union with nature and the universe.

Several studies used Cloninger’s model to trace temperament and character dimensions in psychopathology. For instance, adolescents engaging in NSSI, both patients and non-clinical controls, are characterised by high novelty seeking and low persistence [20,34,35,36]. High NS and low PS both indicate high impulsivity, which could partially explain why those who engage in NSSI struggle with resisting the urge to self-injure [37]. Nock and Prinstein [38] corroborated the impulsive nature of NSSI and found that many individuals contemplated the act of self-injury for less than five minutes before committing it. High NS and impulsivity are linked to low self-directedness, which is also part of the personality profile of NSSI [39,40]. Those with low SD are more likely to experience emotion regulation difficulties and end up losing executive functions when they encounter a negative affect [40]. These individuals tend to have lower emotional clarity and are less likely to accept their own emotions [41]. The lack of adaptive emotion regulation strategies might push individuals with low SD towards NSSI once they are confronted with an overwhelming negative affect [42]. Contributing to these emotion regulation difficulties are the high harm avoidance levels in the personality profile of NSSI [34]. According to Cloninger, the combination of high NS and high HA results in an approach-avoidance conflict that can cause further affective instability [29]. Results are less clear regarding levels of cooperativeness in those engaging in NSSI: the scores seem to be dependent on the absence or presence of suicidal thoughts and behaviours (STBs) [43]. Specifically, female adolescents who engaged in self-injury and experienced STBs showed higher CO scores compared to those without STBs [43]. So far, no associations have been found between reward dependence and NSSI [34,36].

The temperament and character dimensions of EDs are similar to those of NSSI in terms of self-directedness and harm avoidance. Namely, like NSSI, all ED types are characterised by high harm avoidance [44,45,46,47] and low self-directedness when compared to healthy controls [44,45,47]. Within ED subtypes, it was found that individuals with bingeing and purging behaviour (i.e., AN-BP and BN patients) reached even lower SD levels compared to those restricting their food intake (i.e., AN-R patients [46]). Similar to those engaging in NSSI, individuals with bingeing and purging behaviour show high novelty seeking [44,45,46,47] and low persistence [44,46,47]. Thus, the tendency for impulsivity and excitableness is common in both NSSI and binge/purge EDs. However, those with restrictive eating behaviour show the exact opposite pattern: low novelty seeking and high persistence. This combination of characteristics is associated with perfectionism, obsessiveness, and rigidity, which may maintain restrictive, calculated eating behaviour [44,45,46,47]. In contrast to NSSI, low cooperativeness is generally found in all ED types [44,45,46,47]. Finally, although high reward dependence was hypothesised to be a core characteristic of EDs [48], no associations have been found between reward dependence and ED [49,50].

The vast majority of research on EDs and NSSI has been conducted in adult and/or community samples [9,21]. However, the onset of EDs and NSSI occurs during adolescence and prevalence rates peak during these teenage years, particularly so in clinical samples [7,12,13]. Furthermore, little attention has been given to temperament and character traits in adolescent ED patients, be it with or without NSSI, even though previous research has suggested temperament to be a potential transdiagnostic mechanism in EDs and NSSI [20,28]. Therefore, the current study will investigate temperament and character traits in a large sample of young patients diagnosed with an ED and with(out) NSSI. Our first aim is to investigate the frequency and methods of lifetime and recent NSSI across ED subtypes in this sample. Based on previous literature, we expect higher frequencies of lifetime and recent NSSI in ED patients of the bingeing/purging subtype, compared to the restrictive subtype [19,20,21]. Research is lacking regarding NSSI methods, which leaves us unable to make strong hypotheses on frequencies of NSSI methods across ED subtypes. As a second aim, we will examine temperament and character dimensions in the adolescent ED patients with and without NSSI. Based on the literature summarised above, we hypothesise both ED types to show high harm avoidance, low self-directedness and low cooperativeness. Furthermore, we hypothesise a combination of high novelty seeking and low persistence in patients with a bingeing/purging ED and the opposite combination, low novelty seeking and high persistence, in patients with a restrictive ED [44,45,46,47]. If patients are engaging in NSSI, we hypothesise those individuals to be characterised by high novelty seeking, low persistence, low self-directedness, and high harm avoidance [20,34,35,36].

## 2. Materials and Methods

### 2.1. Procedure and Participants.

Between 2011 and 2018, data was collected in female patients who were hospitalised at a specialised inpatient eating disorder ward in Belgium. Shortly after admission, all patients were invited to participate in the study. Those willing to participate provided written informed consent and completed a series of online questionnaires. All patients under 18 years old provided additional parental consent. The procedure of the current study was approved by both the ethical committee of the psychiatric hospital as well as the ethical committee of the faculty of Psychology and Educational Sciences of the first author to use the data retrospectively for research purposes.

The present study included only minor patients who filled out the Eating Disorder Evaluation Scale [51], the Self-Injury Questionnaire-Treatment Related (SIQ-TR [52]) and the brief Dutch version of Cloninger’s Temperament and Character Inventory (VTCI [31]). In total, our sample consisted of 189 female patients with an ED, with a mean age of 15.93 years (SD = 0.98, range 14–17 years).

ED diagnoses, as specified in the DSM-5, were diagnosed by means of an interview conducted by experienced psychiatrists or psychologists and further validated with the Eating Disorder Evaluation Scale [51]. AN-R diagnosis was assigned to 87 participants (46%, M_adjBMI_ = 71.91, SD = 7.32), while 56 participants (29.6%, M_adjBMI_ = 74.32, SD = 6.28) were diagnosed with AN-BP, and 46 (24.3%, M_adjBMI_ = 94.67, SD = 8.16) were diagnosed with BN. Subsequently, data of patients with binge-eating/purging behaviours (AN-BP and BN) were merged, given that these patients show similar temperamental profiles [47]. Thus, we performed our analyses on a group of restrictive ED patients (ED-R; *n* = 87, 46%, M_age_ = 15.91, SD = 0.97) and a group of patients with binge-eating/purging behaviours (ED-BP; *n* = 102, 54%, M_age_ = 15.95, SD = 0.99), with no significant age difference between them (*F*(1, 187) = 0.090, *ns*). Additionally, we used the 13-item depression subscale of the Symptom Checklist-90 (SCL-90-D, [53]) to further describe our sample. Patients with recent NSSI presented with significantly higher depression scores compared to patients without recent NSSI (*F* (1, 188) = 13.957, *p* < 0.000, *η_p_^2^* = 0.070). Similarly, patients with lifetime NSSI presented with significantly higher depression scores compared to patients without lifetime NSSI (*F*(1, 188) = 9.983, *p* = 0.002, *η_p_^2^* = 0.051). ED-BP patients reported significantly higher depression scores compared to ED-R patients when lifetime NSSI was included (*F*(1, 188) = 4.094, *p* = 0.044, *η_p_^2^* = 0.022), but not when recent NSSI was included in the analysis (*F*(1, 188) = 2.265, *p* = 0.134, *η_p_^2^* = 0.012). There was no significant interaction between ED subtype and lifetime/recent NSSI in the prediction of mean depression scores.

### 2.2. Adjusted Body Mass Index (BMI).

We calculated BMI as weight/height^2^ using the weight and height measures provided by the adolescents. Subsequently, we compared the BMI to representative values of adolescent girls in Belgium [54] and computed the adjusted BMI as [(BMI/Percentile 50 of BMI for age and gender) × 100]. The adjusted BMI results in a more accurate determination of the weight status of underaged adolescents and was therefore used throughout this study. In the current sample, 75.1% of the participating patients were underweight (*n* = 142, adjusted BMI ≤ 85) and 24.9% had a normal weight (*n* = 47, 85 < adjusted BMI < 120).

### 2.3. Non-Suicidal Self-Injury

NSSI was assessed using the Self-Injury Questionnaire-Treatment Related (SIQ-TR [55]), a self-report questionnaire that has been proven valid and reliable in female ED patient populations [52]. Patients responded to five yes/no items regarding the absence or presence of scratching, bruising, cutting, burning, and biting oneself. For each self-injurious behaviour, they were asked to complete a number of multiple-choice questions regarding the last time they engaged in that behaviour, how often it happened in certain timeframes, whether they experienced any pain during the behaviour, and which thoughts and feelings preceded and followed the NSSI. Based on these responses, recent NSSI (i.e., any form of NSSI within the last month) and lifetime NSSI (i.e., any form of NSSI throughout their life) were calculated. The Kuder–Richardson reliability coefficient for lifetime NSSI and recent NSSI was 0.673 and 0.489, respectively.

### 2.4. Temperament

The brief Dutch version of Cloninger’s Temperament and Character Inventory (VTCI [31]) was administered to assess Cloninger’s seven temperament scales; novelty seeking (NS), harm avoidance (HA), reward dependence (RD), persistence (PS), self-directedness (SD), cooperativeness (CO), and self-transcendence (ST) [29], [56]. Each of the seven scales contains 15 yes/no items, resulting in a total of 105 items. The VTCI was found to be a reliable and valid instrument in adolescent patient populations [57]. In the present study, Cronbach’s alphas for these seven scales were, respectively, 0.702, 0.763, 0.609, 0.685, 0.806, 0.750, and 0.764.

### 2.5. Statistical Analyses

To analyse the frequency of the different lifetime/recent NSSI behaviours, descriptive statistics were calculated. The associations between the presence/absence of lifetime/recent NSSI and ED subtype (ED-R, ED-BP), were investigated with cross-tabulations and the Chi-square test statistic. Finally, to investigate temperamental differences between ED-R/ED-BP patients with and without lifetime/recent NSSI we performed two separate MANOVAs, both with the VTCI temperament scales as dependent variables and the ED subtype (ED-R, ED-BP) as an independent variable. In the first MANOVA, we included lifetime NSSI as an additional independent variable. In the second MANOVA, we included recent NSSI as an additional independent variable. In both MANOVAs, the interaction between ED and NSSI was included as the third and final independent variable. Partial eta squared (η^2^) was used as a measure of effect size.

## 3. Results

### 3.1. Frequency of Lifetime and Recent NSSI

Overall, 59.8% of the patients (*n* = 113) engaged in at least one act of NSSI during their lifetime whereas 40.2% (*n* = 76) had never engaged in NSSI. The frequency of the reported lifetime NSSI behaviours, when ED-BP and ED-R were examined together, was distributed as follows: cutting (41.8%, *n* = 79), scratching (39.7%, *n* = 75), bruising (27%, *n* = 51), biting (16.9%, *n* = 32), and burning (7.9%, *n* = 15). Table 1 shows the number of different NSSI behaviours (NSSI versatility score) reported by the two ED subtypes. Patients with ED-R reported an average of 2.03 different lifetime NSSI behaviours (SD = 1.13), which was not significantly different from the average of 2.32 different lifetime NSSI behaviours (SD = 1.11) as reported by patients with ED-BP (*F*(1, 112) = 1.741, *p* = 0.190, partial *η*^2^ = 0.015). However, on the level of individual NSSI behaviours, patients with ED-BP reported significantly more lifetime scratching, bruising, cutting, burning, and biting behaviours compared to patients with ED-R (see Table 2).

With respect to recent NSSI, 41.3% of patients (*n = 78*) reported at least one act of NSSI during the last week or the last month. The frequency of the recent NSSI behaviours, when ED-BP and ED-R were examined together, was distributed as follows: 25.4% (*n* = 48) reported scratching, 19.6% (*n* = 37) cutting, 12.7% (*n* = 24) bruising, 7.4% (*n* = 14) biting, and 2.6% (*n* = 5) burning oneself during the last week or month. The number of days one engaged in each of the five NSSI behaviours can be found in Supplementary Table S1. Patients with ED-R reported an average of 1.41 different recent NSSI behaviours (SD = 0.73), which was not significantly different from the average of 1.73 different recent NSSI behaviours (SD = 0.80) reported by patients with ED-BP (*F*(1, 77) = 2.706, *p* = 0.104, partial *η*^2^ = 0.034, see Table 1). However, on the level of individual NSSI behaviours, patients with ED-BP reported significantly more recent scratching, bruising, cutting, and burning behaviours compared to ED-R patients (see Table 2).

The Pearson correlation coefficients between recent/lifetime NSSI and each of the seven temperament and character dimensions can be found in Table 3.

### 3.2. Temperamental Differences between R/BP Patients with(out) Recent NSSI

With temperamental dimensions as the dependent variables, we included ED type, recent NSSI, and the ED × NSSI interaction as the three independent variables in our first MANOVA. Our results showed a main effect of ED subtype, indicating that there was a significant overall difference in temperamental dimensions, based on ED subtype (Wilks’ Lambda = 0.903, *F*(7, 179) = 2.733, *p* = 0.01, partial *η*^2^ = 0.097). As reported in Table 4, the univariate results further clarified that ED-BP patients reported significantly more novelty seeking and less persistence compared to ED-R patients. Second, our results showed a main effect of recent NSSI, indicating a significant overall difference in temperamental dimensions based on presence or absence of recent NSSI (Wilks’ Lambda = 0.88, *F*(7, 179) = 3.478, *p* = 0.002, partial *η*^2^ = 0.12). Namely, the univariate results in Table 4 show how those engaging in recent NSSI reported significantly less novelty seeking, more harm avoidance, and less self-directedness. Finally, there was no significant interaction between ED subtype and recent NSSI (Wilks’ Lambda = 0.985, *F*(7, 179) = 0.392, *p* = 0.906, partial *η*^2^ = 0.015) in the prediction of temperamental differences. To control for age, we conducted an additional MANCOVA with temperamental dimensions as the dependent variables, ED subtype, recent NSSI and the ED × NSSI interaction as independent variables, and age as a control variable. Age did not reach significance: Wilks’ Lambda = 0.974, *F*(7, 179) = 0.682, *p* = 0.687, partial *η*^2^ = 0.026 and all other results remained the same (i.e., main effect of ED subtype, main effect of recent NSSI, no interaction effect of ED subtype × NSSI).

### 3.3. Temperamental Differences between R/BP Patients with(out) Lifetime NSSI

In a second MANOVA, we substituted recent NSSI for lifetime NSSI while the other variables remained the same as in the first MANOVA. Thus, temperamental dimensions remained included as the dependent variables, and ED subtype, recent NSSI, and the ED × NSSI interaction term were included as the three independent variables. First, the main effect of ED subtype did not reach significance (Wilks’ Lambda = 0.93, *F*(7, 179) = 1.89, *p* = 0.073, partial *η*^2^ = 0.069). This lack of significance could potentially be ascribed to a statistical artefact. Specifically, by substituting one independent variable with another, the degrees of freedom and the level of explained variance might deviate slightly, which can result in a different main effect. For completeness, we did still include the univariate level in Table 5. Second, our results showed a main effect of NSSI, indicating a significant overall difference in temperamental dimensions based on the presence or absence of lifetime NSSI (Wilks’ Lambda = 0.921, *F*(7, 179) = 2.198, *p* = 0.036, partial *η*^2^ = 0.079). As described in Table 5, the univariate results clarified that those who engaged in lifetime NSSI reported significantly more harm avoidance and less self-directedness compared to those without lifetime NSSI. Contrasting our findings with recent NSSI, there was no significant difference in the level of novelty seeking between those with and without lifetime NSSI. Finally, the results did not show a significant interaction between ED subtype and lifetime NSSI (Wilks’ Lambda = 0.98, *F*(7, 179) = 0.49, *p* = 0.838, partial *η*^2^ = 0.019), indicating that the association between temperament and NSSI is similar in both ED subtypes. To control for age, we conducted an additional MANCOVA with temperamental dimensions as the dependent variables, ED subtype, lifetime NSSI and the ED × NSSI interaction as independent variables, and age as a control variable. Age did not reach significance: Wilks’ Lambda = 0.974, *F*(7, 179) = 0.667, *p* = 0.700, partial *η*^2^ = 0.026 and all other results remained the same (i.e., main effect of ED subtype, main effect of lifetime NSSI, no interaction effect of ED subtype × NSSI).

## 4. Discussion

The prevalence rates of NSSI in patients with any ED are alarmingly high. Both EDs and NSSI are driven by certain temperamental vulnerabilities, which, in their turn, increase the risk for later personality disorders [58,59]. To improve intervention of EDs and NSSI and prevention of later personality disorders, it is crucial to develop a thorough understanding of transdiagnostic temperamental vulnerabilities in adolescents. The vast majority of research on temperamental dimensions in EDs and NSSI has been conducted in adult samples [9,21]. Therefore, the current study examined temperament and character dimensions in a sample of adolescent ED patients with and without NSSI.

As a first aim, the present study investigated the prevalence of NSSI as well as the methods used to engage in NSSI. Our results confirmed the alarmingly high prevalence rates of NSSI previously found in young ED patients [16]. Namely, 60% of the current sample reported lifetime NSSI (i.e., having ever engaged in NSSI) and 40% of the sample reported recent NSSI (i.e., having engaged in NSSI in the past month). Thus, as this information was collected shortly after admission to an inpatient treatment facility, the latter indicates that 40% of the adolescents engaged in NSSI right before and/or during their ED treatment at the hospital ward. Interestingly, previous phenomenological qualitative research described how, during treatment, individuals with an ED experienced a loss of control when they were pressured to eat [60]. To lose control in one domain, be it due to treatment or due to pressure by parents or peers, often requires compensation in another domain [61]. Indeed, the participants in the phenomenological study reported how NSSI functioned as a means of control over anorectic thoughts and overwhelming emotions when they felt pressured to eat [60]. Future systematic research could investigate whether the high prevalence rates of NSSI found in the current study could be due to patients attempting to regain a sense of control.

Our results confirmed cutting and scratching to be the most common forms of NSSI, with, respectively, 42% and 40% of ED patients having engaged in these behaviours [16]. Moreover, each of the five assessed NSSI behaviours was significantly more common in patients of the ED-BP subtype, compared to the ED-R subtype. This ED-R/ED-BP distinction remained unchanged whether lifetime NSSI behaviours or recent NSSI behaviours were assessed. These findings align with previous research, which indicated that ED patients with bingeing/purging symptomatology engaged more often in NSSI overall [19,20,21]. Only for “biting oneself in the last month” the difference between ED-R and ED-BP did not reach significance, possibly due to the very low prevalence rate of “biting oneself” in both groups. Previously, research was lacking in how the specific NSSI behaviours compared between ED subtypes. With only four previous studies available, our results innovate by providing evidence for higher lifetime prevalence of cutting, scratching, bruising, biting, and burning in ED-BP compared to ED-R patients.

In search of a transdiagnostic mechanism in the ED–NSSI interplay, the current study investigated temperament and character dimensions as a second aim. First, our results showed significant differences in temperament and character based on ED subtype. Namely, patients with ED-BP showed significantly more novelty seeking and less persistence compared to patients with ED-R. Thus, the ED-BP patients had a greater tendency to seek out new, exciting experiences, possibly making impulsive decisions while doing so (high novelty seeking). Additionally, they were less likely than the ED-R patients to persevere and overcome setbacks or frustration, but rather felt frustrated or overwhelmed (low persistence). The differences between ED-BP and ED-R patients as found in the present study are consistent with ED literature [62,63,64,65]. Furthermore, both high novelty seeking and low persistence indicate impulse dysregulation, which previous research indeed attributed to bingeing and purging behaviours in ED patients [38]. Importantly, because NSSI too is characterised by high novelty seeking and low persistence [20,34,35,36], this temperamental profile might function as a transdiagnostic mechanism explaining the high comorbidity between NSSI and ED-BP specifically.

Second, our results showed significant differences in temperament and character based on the presence or absence of NSSI, regardless of the ED subtype. Specifically, ED patients with recent and/or lifetime NSSI reported less self-directedness and more harm avoidance compared to patients who were not engaging in NSSI. Thus, ED patients engaging in NSSI experience less control over their own emotions, they struggle to regulate themselves to set and reach goals (low self-directedness). They lack emotion regulation skills in comparison to adolescent ED patients who do not engage in NSSI. Moreover, NSSI in ED patients was associated with high harm avoidance, which indicates shyness and anxiety in social situations as well as excessive worrying and insecurity regarding interactions with others. These findings align with previous research indicating that emotion dysregulation and an increased amount of negative thoughts and feelings are common in those engaging in NSSI [5,61,66]. Specifically in ED patient populations, high harm avoidance can be related to self-punishment and rumination for those who self-injure. Patients with an ED who additionally engage in NSSI tend to be more concerned about meeting expectations of themselves and others, compared to patients with and ED who do not engage in NSSI [67]. In a qualitative study, ED patients in treatment facilities described how they would use NSSI to punish themselves when they failed to meet their own standards or felt that they had disappointed healthcare workers [60]. In conclusion, the temperamental profile of low self-directedness and high harm avoidance characterises patients with any ED who engage in NSSI.

Interestingly, when studying temperament in ED patients with recent NSSI, one more dimension besides low self-directedness and high harm avoidance showed up as significantly different in those with and without recent NSSI, regardless of ED subtype. Namely, ED patients engaging in recent NSSI reported significantly less novelty seeking. This stands for less curiosity and less impulsivity in those with recent NSSI, as they were less likely to seek out or be interested in new experiences, compared to ED patients without recent NSSI. Previous research, however, typically reported high levels of novelty seeking in adolescent patients engaging in NSSI [20,34,35,36]. Remarkably, these studies generally assessed lifetime, rather than recent, NSSI. A tentative suggestion as to why we found low novelty seeking in those with recent NSSI, could be the comorbidity of NSSI with depression at the time of assessment.

Depression is typified by high harm avoidance and low self-directedness and, importantly, low novelty seeking; a lack of interest, enthusiasm, and curiosity in one’s surroundings [68]. At the time of assessment, those engaging in recent NSSI might have presented with more outspoken depressive symptoms, resulting in lower levels of novelty seeking compared to those without recent NSSI. Previous studies have indeed shown that at NSSI onset, impulsivity and high novelty seeking are common [34,69]. When individuals first start engaging in NSSI, often by experimenting once or twice with a certain method of self-injury, they are more likely to be looking for sensation or a new experience. Consequently, studies investigating lifetime NSSI report high novelty seeking [20,34,35,36]. However, research on various addictive behaviours suggested that, once the behaviour goes beyond mere experimenting and becomes a coping strategy in response to psychiatric distress, the impulsive, sensation-seeking function loses ground [70]. Rather, the emotion regulation function might become more salient as those with severe, persistent addiction(s) and comorbid psychopathology report engaging in the behaviour to avoid and regulate overwhelming negative affect [70]. Although future research should assess if this parallel with research on addiction is justified, our results on NSSI in ED patients do suggest a similar pattern. Namely, ED patients who present at a treatment facility with recent NSSI show temperamental and character dimensions resembling depression: low self-directedness, high harm avoidance, and, contrasting previous research, low novelty seeking.

The results of the current study support the focus on emotion and impulse dysregulation in evidence-based treatment of NSSI in ED patients. For instance, Dialectical Behavioural Therapy [71] and the Cutting Down treatment programme [72] have been proven effective in this specific patient population. High levels of harm avoidance could be related to serotonergic dysfunction [73] and could, therefore, be targeted by pharmacological treatments, for instance by the use of SSRI to focus on binge eating and self-harm in depressed ED patients [73]. Low levels of self-directedness could be treated by executive function training (e.g., by means of the “Playmancer” computer game [74]).

Although the present study contributes to the understanding of temperament ED patients with and without NSSI, our research is not without limitations. First, our findings are based solely on adolescent self-report questionnaires. Collecting self-report data from a single piece of information could result in reporting bias [75]. However, while we could have assessed peers, parents, or teachers about NSSI and its correlates, research has shown that people do not always observe internalising behaviours accurately in others [76], making NSSI and temperament often difficult to assess by other informants. Additionally, NSSI is often secretive [77] and people close to the adolescent who engages in NSSI, such as parents, are often unaware of the presence or severity of the NSSI [78]. Future research could embrace a multi-method approach and include structured or semi-structured interviews with the adolescents and/or use behavioural measures. Second, because the present study sample solely consists of adolescent girls in an eating disorder treatment facility, we cannot generalise the reported findings to male populations, younger or older individuals, or those receiving ambulatory care. Third, our conclusions are restricted by the cross-sectional nature of our study. As the field moves forward, longitudinal studies will be necessary to examine the developmental course and directionality of effects of temperament in ED patients with and without NSSI.

## Figures and Tables

**Table 1:** The number of different lifetime and recent NSSI behaviours ED-R and ED-BP engaged in.

	Lifetime NSSI *n* = 113				Recent NSSI *n = 78*			
	ED-R *n* = 36		ED-BP *n* = 77		ED-R *n* = 22		ED-BP *n* = 56	
	% ^a^	*n*	% ^a^	*n*	% ^a^	*n*	% ^a^	*n*
1 behaviour	38.9%	14	28.6%	22	68.2%	15	44.6%	25
2 behaviours	36.1%	13	29.9%	23	27.3%	6	41.1%	23
3 behaviours	13.9%	5	23.4%	18	0%	0	10.7%	6
4 behaviours	5.6%	2	16.9%	13	4.5%	1	3.6%	2
5 behaviours	5.6%	2	1.3%	1	0%	0	0%	0

Note. ^a^ Percentages of NSSI behaviours within ED category; NSSI = Non-Suicidal Self-Injury; ED-R = eating disorder of the restrictive type; ED-BP = eating disorder of the bingeing/purging type.

**Table 2:** Presence and absence of five NSSI behaviours in two ED subtypes.

	ED-R *n* = 87				ED-BP *n* = 102				
	Present		Absent		Present		Absent		
	% ^a^	*n*	% ^a^	*n*	% ^a^	*n*	% ^a^	*n*	*Χ* ^2^_(1)_
**Lifetime**									
Scratch	28.7%	25	71.3%	62	49.0%	50	51.0%	52	8.071 **
Cut	24.1%	21	75.9%	66	56.9%	58	43.1%	44	20.669 ***
Bruise	17.2%	15	82.8%	72	35.3%	36	64.7%	66	7.766 **
Bite	10.3%	9	89.7%	78	22.5%	23	77.5%	79	4.972 *
Burn	3.4%	3	96.6%	84	11.8%	12	88.2%	90	4.444 *
**Recent**									
Scratch	12.6%	11	87.4%	76	36.3%	37	63.7%	65	13.838 ***
Cut	11.5%	10	88.5%	77	26.5%	27	73.5%	75	6.689 **
Bruise	4.6%	4	95.4%	83	19.6%	20	80.4%	82	9.542 **
Bite	6.9%	6	93.1%	81	7.8%	8	92.2%	94	0.061
Burn	0%	0	100%	87	4.9%	5	95.1%	97	4.381 *

Note. * *p* < 0.05, ** *p* < 0.01, *** *p* < .001; ED-R = Eating Disorder, Restrictive Subtype; ED-BP = Eating Disorder, Binge Eating/Purging Subtype, NSSI = Non-Suicidal Self-Injury. ^a^ Percentages of absent/present NSSI behaviour within each ED subtype.

**Table 3:** Correlation coefficients between study variables.

	Lifetime NSSI	Recent NSSI	NS	HA	RD	PS	SD	CO
Recent NSSI	0.687 ***							
NS	–0.016	–0.118						
HA	0.205 **	0.252 ***	–0.370 ***					
RD	–0.098	–0.065	0.118	–0.203 **				
PS	–0.049	0.084	–0.345 ***	–0.024	0.121			
SD	–0.225 **	–0.204 **	0.108	–0.488 ***	0.130	0.089		
CO	0.043	0.103	–0.242 **	0.026	–0.214 **	–0.232 **	–0.213 **	
ST	0.093	0.087	0.089	–0.060	0.102	–0.095	–0.185 *	–0.071

Note. * *p* < .05, ** *p* < .01, *** *p* < .001; Significant correlation coefficients are marked in bold; NS = Novelty Seeking, HA = Harm Avoidance, RD = Reward Dependence, PS = Persistence, SD = Self-Directedness, CO = Cooperativeness, ST = Self-Transcendence.

**Table 4:** Means and standard deviations of the *z*-scores of the brief Dutch version of Cloninger’s Temperament and Character Inventory for ED-R/BP patients with(out) recent NSSI (last week/last month).

	Main Effect ED Subtype					Main Effect Absence/Presence of NSSI_R_				
	ED-R *n = 87*		ED-BP *n = 102*			NSSI_R_ = 0 *n = 111*		NSSI_R_ = 1 *n = 78*		
	*M*	*SD*	*M*	*SD*	*F(1, 185)*	*M*	*SD*	*M*	*SD*	*F(1, 185)*
NS	–0.25	(0.91)	0.24	(1.04)	18.47 ***	0.11	(1.02)	–0.13	(0.98)	9.04 **
HA	–0.00	(0.96)	–0.00	(1.02)	1.54	–0.21	(1.03)	0.29	(0.85)	14.23 ***
RD	0.02	(1.04)	–0.01	(0.95)	0.02	0.06	(1.07)	–0.07	(0.86)	0.78
PS	0.14	(.78)	–0.12	(1.15)	4.45 *	–0.07	(1.04)	0.10	(0.95)	2.94
SD	0.09	(1.12)	–0.11	(0.90)	0.03	0.16	(1.11)	–0.26	(0.78)	7.70 **
CO	0.07	(1.09)	–0.07	(0.92)	1.76	–0.09	(1.06)	0.12	(0.91)	2.75
ST	–0.12	(1.01)	0.08	(1.01)	1.08	–0.08	(0.93)	0.09	(1.11)	0.57

Note. * *p* < 0.05, ** *p* < 0.01, *** *p* < 0.001; ED-R = Eating Disorder, Restrictive Subtype; ED-BP = Eating Disorder, Binge Eating/Purging Subtype, NSSI_R_ = Recent Non-Suicidal Self-Injury; NS = Novelty Seeking, HA = Harm Avoidance, RD = Reward Dependence, PS = Persistence, SD = Self-Directedness, CO = Cooperativeness, ST = Self-Transcendence.

**Table 5:** Means and standard deviations of the *z*-scores of the brief Dutch version of Cloninger’s Temperament and Character Inventory for ED-R/BP patients with(out) lifetime NSSI.

	Main Effect ED Subtype					Main Effect Absence/Presence of NSSI_L_				
	ED-R *n = 87*		ED-BP *n = 102*			NSSI_L_ = 0 *n = 76*		NSSI_L_ = 1 *n = 113*		
	*M*	*SD*	*M*	*SD*	*F(1, 185)*1	*M*	*SD*	*M*	*SD*	*F(1, 185)*
NS	–0.25	(0.91)	0.24	(1.04)	12.68 ***	0.03	(0.99)	0.00	(1.03)	2.16
HA	0.00	(0.96)	0.00	(1.02)	0.97	–0.25	(0.97)	0.16	(0.98)	9.19 **
RD	0.02	(1.04)	–0.01	(0.95)	0.07	0.12	(1.01)	–0.07	(0.97)	1.82
PS	0.14	(0.78)	–0.12	(115)	1.87	0.06	(0.87)	–0.04	(1.09)	0.02
SD	0.09	(1.12)	–0.11	(0.90)	0.20	0.26	(1.16)	–0.20	(0.84)	7.82 **
CO	0.08	(1.08)	–0.08	(0.92)	1.92	–0.06	(1.05)	0.03	(0.98)	1.06
ST	–0.12	(1.01)	0.08	(1.01)	0.63	–0.12	(0.93)	0.07	(1.06)	0.82

Note. ** *p* < 0.01, *** *p* < 0.001; ED-R = Eating Disorder, Restrictive Subtype; ED-BP = Eating Disorder, Binge Eating/Purging Subtype, NSSI_L_ = Lifetime Non-Suicidal Self-Injury; NS = Novelty Seeking, HA = Harm Avoidance, RD = Reward Dependence, PS = Persistence, SD = Self-Directedness, CO = Cooperativeness, ST = Self-Transcendence.

## Supplementary Materials

The following are available online at https://www.mdpi.com/2077-0383/9/4/1207/s1, Table S1: Number of days individuals engaged in five NSSI behaviours in the last month.
