# Supplementary material for: Temperament and Character Traits of Female Eating Disorder Patients with(out) Non-Suicidal Self-Injury
Source: J Clin Med. 2020 Apr 22;9(4):1207. doi: 10.3390/jcm9041207 (PMC7230745; doi:10.3390/jcm9041207)
Supplement: Supplementary file 1 [file jcm-09-01207-s001.pdf]

Supplementary Table S1. Number of days individuals engaged in five NSSI behaviours in the last month.

	Scratch *n* = 48				Bruise *n* = 24				Cut *n* = 37				Burn *n* = 5				Bite *n* = 14			
	ED-R *n* = 11		ED-BP *n* = 37		ED-R *n* = 4		ED-BP *n* = 20		ED-R *n* = 10		ED-BP *n* = 27		ED-R *n* = 0		ED-BP *n* = 5		ED-R *n* = 6		ED-BP *n* = 8	
	% [a]	*n*	% [a]	*n*	% [a]	*n*	% [a]	*n*	% [a]	*n*	% [a]	*n*	% [a]	*n*	% [a]	*n*	% [a]	*n*	% [a]	*n*
1–5 days	54.5%	6	67.6%	25	75%	3	75%	15	70%	7	51.9%	14		0	100%	5	66.7%	4	75%	6
6–10 days	27.3%	3	8.1%	3	0%	0	5%	1	10%	1	22.2%	6		0	0%	0	16.7%	1	12.5%	1
11–15 days	18.2%	2	8.1%	3	0%	0	15%	3	10%	1	7.4%	2		0	0%	0	0%	0	12.5%	1
>15 days	0%	0	16.2%	6	25%	1	5%	1	10%	1	18.5%	5		0	0%	0	16.7%	1	0%	0

Note. [a] Percentages of NSSI frequency per method within ED category; ED-R = eating disorder of the restrictive type; ED-BP = eating disorder of the bingeing/purging type.
